# Water, hygiene and sanitation practices are associated with stunting among children of age 24-59 months in Lemo district, South Ethiopia, in 2021: community based cross sectional study

**DOI:** 10.1186/s40795-023-00677-1

**Published:** 2023-01-23

**Authors:** Biruk Woldesenbet, Alemu Tolcha, Berhan Tsegaye

**Affiliations:** 1 Department of Environmental Health Science, College of Medicine and Health Sciences, Hawassa University, Hawassa, Ethiopia; 2 Department of Midwifery, College of Medicine and Health science, Hawassa University, Hawassa, Ethiopia

**Keywords:** Water, Sanitation, Hygiene, Stunting, Children, Food insecure

## Abstract

**Background:**

Stunting among children of ages 24-59 months is a major public health challenge in developing countries. It has been linked with poor water quality, water accessibility, a lack of environmental sanitation, and personal hygiene (WASH) practices, particularly in food-insecure areas. Stunting occurs during certain seasons in food-insecure settings. Therefore, a complete understanding of risk factors is the first step in the development of a preventive strategy. However, information is scarce about the prevalence and factors associated with stunting among children of ages 24-59 months in these settings.

**Objective:**

This study aimed to assess the prevalence of and factors associated with stunting among children aged 24–59 months in Lemo district, south Ethiopia, in 2021.

**Methods:**

A community based cross-sectional study was conducted from January 1-30/2021. Data were collected from a total of 415 randomly selected children and mother /guardian/. Logistic regression analysis was done to identify factors associated with childhood stunting. In binary logistic regression analysis, independent variables with *p*-value < 0.25 were fitted into multivariable logistic regression analysis to explore final predictors of stunting/ thinness/. Independent variables with AOR and 95% CI and *P*-value < 0.05 was computed and reported as predictors of stunting among children in this study.

**Results:**

From a total of 450 children, only 415 were included in the final analysis, making a response rate of 92.2%. The prevalence of stunting among children was 33.5% (95% CI: 30.4 and 36.6%) in this study. Children ages 48-59 months (AOR = 2.8, 95% CI: 2.1, 12.1), children ages 36-47 months (AOR = 1.6, 95% CI: 1.1, 7.1), children of uneducated women (AOR = 1.8, 95% CI: 1.5, 4.2), children who lived near unimproved toilets (AOR = 1.7, 95% CI: 1.2, 2.6), children whose feces was disposed of unsafely (AOR = 2.8, 95% CI: 1.57, 5.31), and children whose mothers did not wash their hands before feeding their children (AOR = 6.2, 95% CI: 2.0, 19.1) were factors positively associated with stunting among children aged 24 months to 59 months.

**Conclusion:**

The prevalence of stunting is high compared with the national prevalence of stunting in food insecure areas. Policy makers, local leaders, and community health extension workers should enhance environmental sanitation and create awareness about personal hygiene. Furthermore, improved toilet construction and appropriate utilization should be encouraged. Furthermore, the local government should work to improve the socio-economic status of poor households.

## Background

Nutritional status of children is the key aspect of children growth and development because it determines their future mental health, physical growth and maturity, and academic performance throughout their life. Globally, it is responsible for more than one-third of under-five children deaths. One every five children of under five years are stunted [1]. Abnormal growth of body of children is mainly irreversible in human capital development [2, 3].

Stunting (low height-for-age) is the chronic restriction of a child’s potential growth. It refers to children from the ages of 24 to 59 months who are below 2-standard deviations from the median height-for-age determined by the World Health Organization (WHO) child growth standards [4, 5]. Although stunting among preschool children had been declined in some regions of Asia from 1990 to 2015, it remains stagnant in Sub-Saharan Africa countrie s[6].

Food insecurity is lack of power to access safe and adequate food to meet their daily demands [7, 8]. Food insecurity causes hanger and acute malnutrition and their complications [9]. Generally, more than half of all children mortality was associated with under-nutrition [10]. Based on the report of 2019 demographic and health survey of Ethiopian, 38% under-five children were stunted in Ethiopia [11]. Ethiopia has demonstrated promising progress in reducing under-nutrition over the past decade. However, prevalence of stunting has been hardly decreased [12]. Water, hygiene and sanitation practices are fundamental human rights [13]. However, stunting is linked with WASH practices through multiple mechanisms such as repeated diarrhoea, infection pathways, and environmental enteric dysfunction [14]. For example, pure water is scarce among rural communities for domestic purpose and majorities of them rely on surface water for daily consumptions which can impose them for many health hazard since it is untreated and contaminated [11]. Moreover, surface water is shared among human, domestic animal, and wild animals. Water treatment methods such as: Boiling, ffiltration and disinfection are not yet implemented due to financial and cultural barriers especially in rural Ethiopia. Furthermore, open defecation is still practiced in most communities and only few of them owned pit latrines without slab [11]. Moreover, open defecation poses a risk of fecal contamination during defecation [10].

A number of environmental, social, demographic, cultural, and economic factors contribute to poor nutritional status. Specifically, stunting among children of under-five years was associated with these factors since they are ecologically linked with each other [15, 16].

The indirect causes of stunting include: Food insecurity, poor childcare practices, low maternal educational status, and lack of access to health services, clean water, sanitation, and poor hygiene practice [17, 18]. Pre-school children are vulnerable segment the population stunting. Stunting is one of the sensitive indicators of community health and nutrition. Previous study reported that most (64.1%) of the households in Lemo district had poor sanitation facilities [19]. Based on the report of previous study, only 36% of the population who lived in the current study area is approved to be food secure. Specifically, 18, 13 and 33% of the population lived in mild, moderately and severely food in secure setting respectively [20]. Therefore, this study aimed to assess prevalence and factors associated with stunting among children aged 24-59 months in Lemo Districts, South Ethiopia, in 2022.

## Methods

### Study design

Hadiya zone is located in Ethiopia’s Southern Nation Nationalities and Peoples Regional States (SNNPR). It is divided into ten districts and one city administration. It is located at a distance of 230 km to the northeast of Addis Ababa, the capital city of Ethiopia. Lemo is one of the food-insecure districts in Hadiya Zone. Geographically, it is situated between 7°22′00″–7°45′00″N latitude and 37°40′00″–38°00′00″E longitude. According to the 2007 Central Statistical Agency (CSA) report, the total population of Lemo district is 118,594. It is composed of 58,666 males and 59,928 females. Most (98.3%) of the population lives in rural settings. Consequently, most (65.5%) of the population in the district are farmers. Enset-based mixed crop-livestock production is the main agricultural production system. According to the annual report of the Lemo district health office, the estimated number of preschool children whose age was accounted for was 16,335 in 2018. Preschool children comprise 10.4% of the total population in the district. There are seven health centres and 35 health posts in the district that provide malnutrition diagnosis and treatment at the time of data collection. In addition, 65 health extension workers work in health posts.

### Population

The target population consisted of children aged 24 to 59 months who lived in the Lemo district. Children of age 24-59 months who lived in randomly selected households and presented during the data collection period were the study population in this study. On the contrary, mothers, guardians, or children who were unable to give a full response or were incompetent for anthropometric measurement due to critical illness during the data collection period were excluded from the study.

### Sampling and sample size determination

**Fig. 1:**
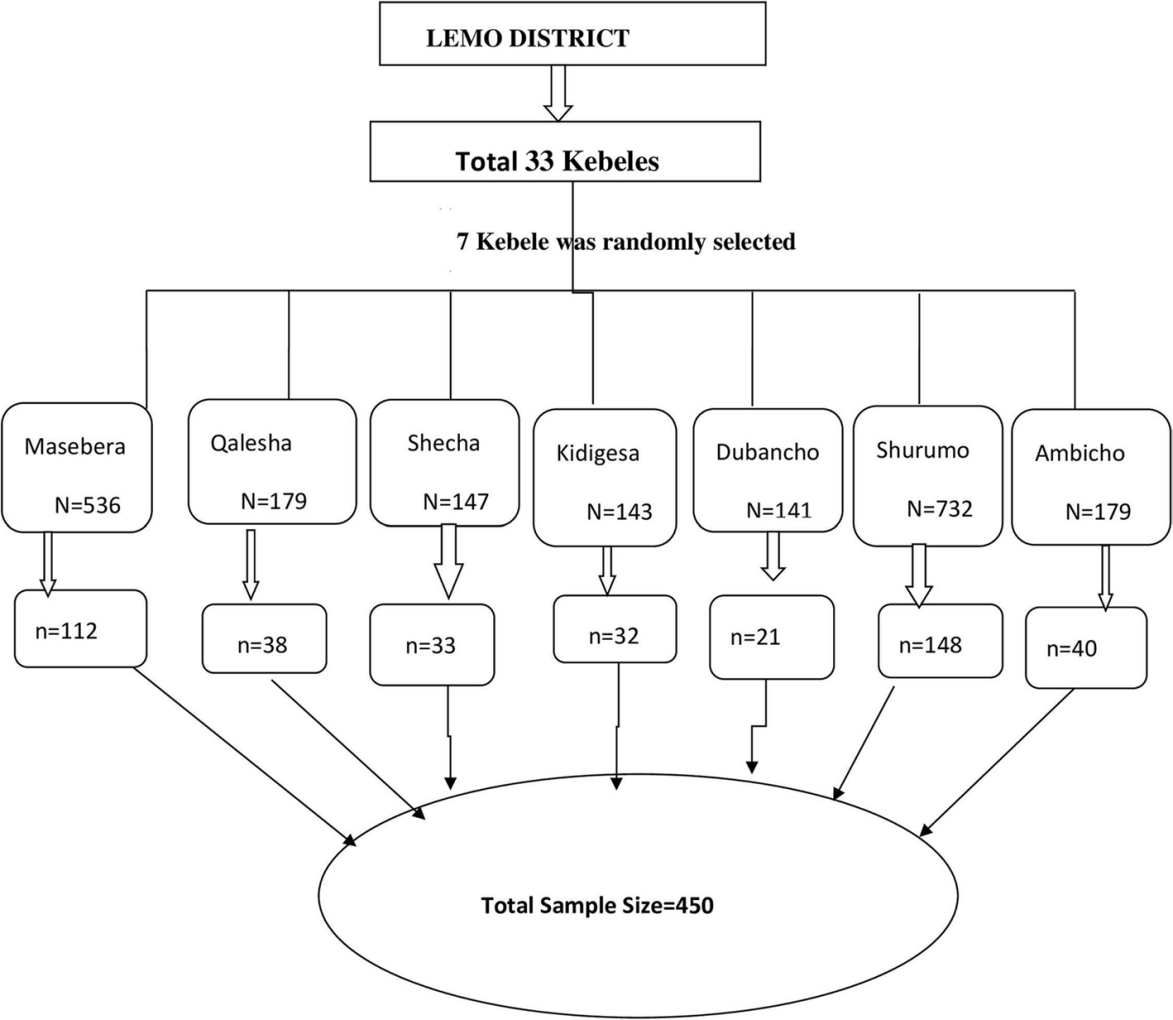
Schematic presentation of sampling procedure of studnting among preschool children in Lemo district,Hadiya,South Ethiopia,in 2021.

The sample size was determined using the single population proportion formula, taking the following assumptions: Stunting prevalence among children (*p* = 23.1%) from an Ethiopian meta-analysis study [21], 95% confidence level, 5% margin of error, design effect of 1.5, and 10% non-response rate. Hence, the initial sample size was computed using the standard Cochran formula, *n* = z2pq/d2. Then, plugging values into the formula, a total sample of 273 was computed. Using sample adjustment formulas (a design effect of 1.5 and a non-response rate of 10%), the final sample size was calculated as 450. Figure 1 displays the sampling strategy of this study. A multistage stratified cluster sampling technique was applied to select study samples in this study. In the first stage, 1 urban Kebele and 10 rural Kebele were selected using simple random sampling, following the technique of the lottery method. In the second stage, households were selected using a systematic random sampling technique. For this study, the health posts’ family folder (or “registration book”) was utilized as a sampling frame to select households that consist of children aged 24-59 months. We have utilized the updated registration book of health extension workers who gave service in the health posts, which consists of the name of the Kebele and the house number of children ages 24-59 months. The first household was identified using the lottery method (simple random sampling). Then, subsequent households were selected for every K-value (K = N/n) to reach the selected households. Where N is the total number of households in the selected Kebeles and n is the calculated sample size.

Figure 1 showed the schematic presentation of sampling strategy in this study. The sample households selected for this study were allocated proportionally to each Kebele. As a result, we used a different K-value. For households that consisted of more than one child, simple random sampling was used to select the study participants. Furthermore, data collectors visited households that were closed at the time of data collection 2-3 times before declaring them non-responsive. Sample size for the second objective, or assessment of factors associated with stunting, was determined using open-source epi-online software. However, the sample size computed for prevalence was found to be higher than the sample size computed for the second objective. Hence, the sample size for prevalence was considered for this study (See Fig. 1).

### Measurement

**Fig. 2:**
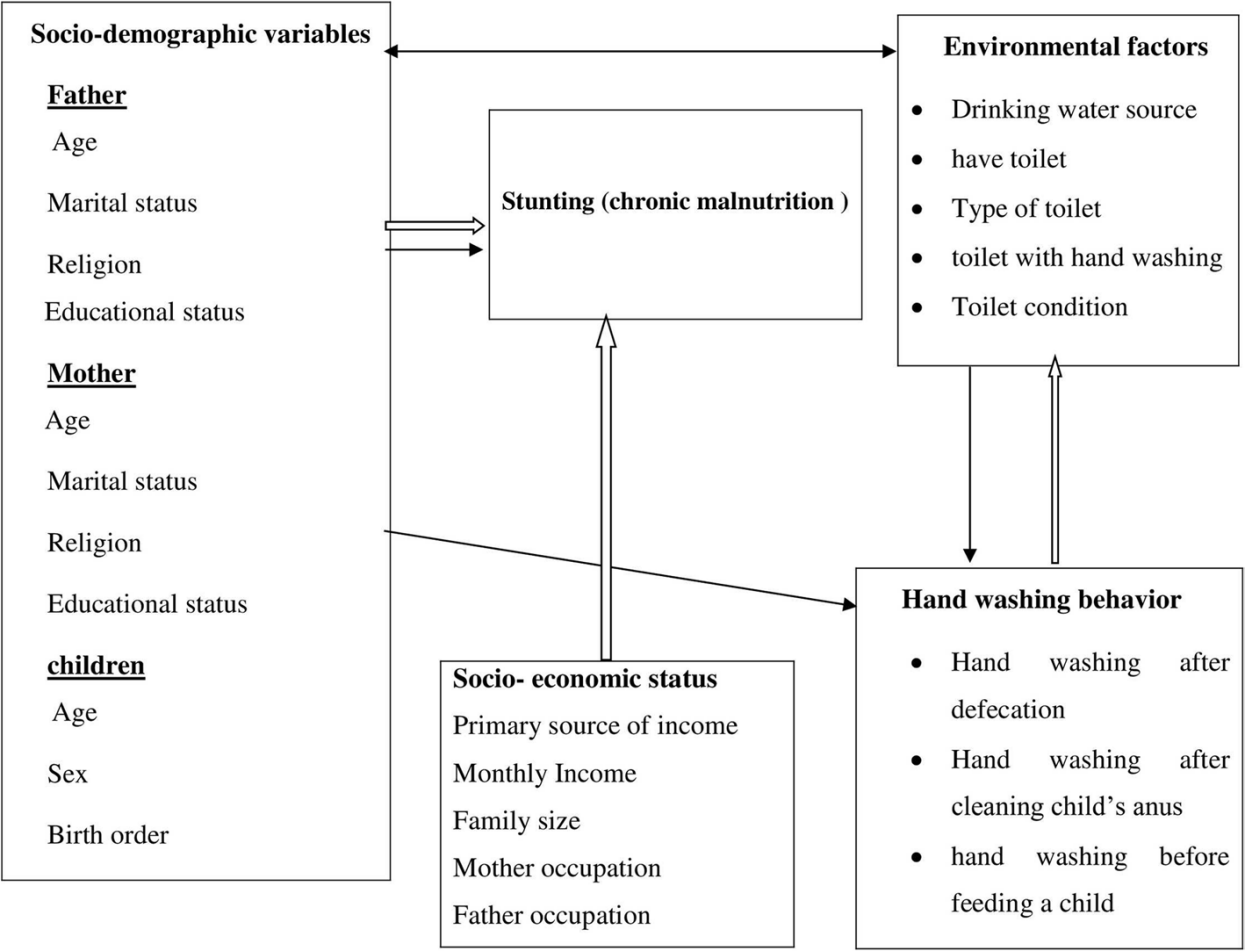
Conceptual framework of effect of water,sanitation and hygiene on stunting among children aged 24-59 months in Lemo district,Hadya,South Ethiopia,2021

The outcome variable of this study was stunting. It is the binary outcome variable which can be determined through anthropometric measurements. Whereas, the explanatory variables include the parental characteristics (both father and mother of children), household level characteristics, child characteristics and health behavior of parents. Figure 2 from this study indicates the complex interaction of independent variables with response variables. For example, the parents’ characteristics include: socio-demographic status, including age, marital status, educational status, religion and income. Furthermore, the child’s characteristics were age and sex of the child. The health behavior factors included the hygiene and environmental sanitation of the parents (See Fig. 2).

Height was measured with a vertical or horizontal measuring board reading a maximum of 175 cm and capable of measuring to 0.1 cm, which was used to take the height of a child. The child stands on the measuring board barefooted, with hands hanging loosely, feet parallel to the body, and heels, buttocks, shoulders, and the back of the head touching the board. The head would be kept comfortably straight, with the lower edge of the eye socket in the same horizontal plane as the outer ear canal. The head of the measuring board is then pushed gently, crushing the hair and coming into contact with the upper part of the head. The height is then computed to within 0.1 cm. Two readings were recorded and the mean calculation was used in the analysis. The length was measured by placing the kid flat on the length board. The slider is placed on the edge of bare feet when the head (crushing the hair) touches the other end of the meter. Then two surveys were carried out, and the average was calculated. The weight was measured using an easily transportable balance measuring 0.1 kg. The ladder was adjusted before weighing each child to zero. The child was lightly dressed when the weight was taken. Two readings were carried out for each child, and the average was entered on the questionnaire [21].

### Operational definitions

#### Food insecurity

 is a condition in which people experience limited or uncertain physical and economic access to safe, sufficient, and nutritious food to meet their dietary needs or food preferences [8].

#### Stunting is a long-term cumulative effect of poor nutrition and poor health

Short stature refers to a low height-for-age ratio that can be caused by either normal growth variation or a growth deficit. It is defined as low height-for-age at − 2 SD below the median value of the WHO international growth reference [21].

### Data collection tools and procedure

A structured and pre-tested questionnaire was used to collect data. The questionnaire was initially written in English. Then, it was translated to Hadyiyissa and back to English to keep its consistency. The questionnaire was prepared through a comprehensive literature review [22–24]. The questionnaire consists of the following parts: Socio-demographic, reproductive, environmental, child health, and care practices A face-to-face interview was conducted in the local language (Hadyiyissa) by data collectors who speak and understand the language. Furthermore, anthropometric measurements were taken to determine the stunting status of children: The height and age of the children were measured. Children of ages 24 to 59 months were examined for signs of stunting by seven bachelor’s degree nurses who had previous experience measuring child malnutrition. The growth standard anthropometric measurement procedure was performed based on the World Health Organization’s recommendation [25].

Interrogation was used to determine children’s ages, which were then confirmed by probing mothers and guardians. Information about the age of children was collected from the health posts’ family folder and the child’s mother or caregiver. A standard calibrated machine was used to record weight in kilograms. Each child’s weight was measured barefoot and without heavy clothing during the measurement process. First, the weight scale was calibrated to zero before taking every measurement. Second, height was measured after the child was placed on the platform, barefooted, with their head upright and looking straight ahead, using a standard height measuring scale. Finally, the weight and height measurements were approximated to the nearest 0.1 value and reported.

### Data quality control

A pre-test was performed on 5% of households that were not included in Kebeles outside of the selected Kebeles. Three days of training were provided to all data collectors and supervisors by the principal investigators. Furthermore, at the end of every data collection day, the completeness of the questionnaires was checked by immediate supervisors, and challenges were discussed and solved for the next day. Inconsistent data were rejected from analysis. We have checked that the reason for the non-responders’ refusal was not linked with the purpose of this study.

### Data analysis

Data were entered, cleaned, and coded using Epi-Info 7 software. Then, it was exported to Statistical Package for Social Sciences (SPSS) software version 25 for analysis. The World Health Organization (WHO) anthro program version 3.2.2 software was utilized to generate the stunting index and export it to SPSS version 25. Before the actual logistic regression analysis, the necessary assumptions of the logistic regression analysis were checked: Errors are independent and exhibit linearity in the logit for continuous variables, absence of multicollinearity, and a lack of strongly influential outliers. WHO growth reference was used to report anthropometric results; individual anthropometric data was compared with reference values on a graph using sex and age-specific Z-score classification systems. The stunting status indicator of height-for-age (HAZ) was compared with the reference data from the World Health Organization standard. A cutoff of below 2 standard deviations (SD) of the WHO median value for HAZ was considered. Frequencies and cross tabulations were used to check for missed values and variables. Descriptive analysis was made using percentages, means, and standard deviations for the variables included in the study. Candidates for the multivariable logistic analysis model were variables with a *P*-value of 0.25. Multivariate logistic regression analysis was used to adjust for possible confounders. To assess the strength of the association, an adjusted odds ratio (AOR) with a 95% confidence interval (CI) and a P-value of 0.05 was calculated. Finally, Hosmer-Lemeshow goodness of fit tests was used to check the model’s fitness.

## Results

### Description of socio-demographic characteristics of study participants

Table 1 presents socio-demographic status of the study participants. It indicates that only 415 of the 450 households visited for this survey received complete responses, for a response rate of 92.2%. Based on the report in this table, the age of mothers /legal guardians/ was computed as 32.7+ 4.7 years. Most (80.2%) of mothers/guardians had no job at the time of data collection. Most (90.6%) of women were protestants. Regarding ethnicity, two out of five women were Hadiya. Nearly two-thirds (69.4%) of surveyed households had five or more members. The majority of households (48.5%) were occupied by farmers in their work. More than half of the households (52.5%) earned less than 1000 Ethiopian birr per month (See Table 1).

**Table. 1:** Children charactestics and socio-demographic factors of mothers/caregivers of children of age 24-59 Months in Lemo district, South Ethiopia, 2021 (*n* = 415)

Variables	Frequency	Percent
**Educational status of Mother**		
Unable to read and write	162	39.0
Can read and write	5	1.2
Primary education	170	41.0
Secondary education	71	17.1
Tertiary education	7	1.7
**Mothers’ occupation**		
House wife	333	80.2
Civil servant	4	1.0
Merchant	70	16.9
Private organization	8	1.9
**Sex of a child**		
Male	204	49.2
Female	211	50.8
**Child’s age in month**		
24-35	107	25.8
36-47	149	35.9
48-59	159	38.3
**Family size**		
Less than five	126	30.4
More than five	289	69.6

### Sex difference of stunting among children of age 24- 59 months

The WHO standard reference took 2 SD as a cutoff point; children who fell below 2 SD were found to be 139 (33.5%) in this study. The prevalence of stunting (height for age below 2% of the median WHO reference values) was higher in female children (53.2%) than their counterparts.

### WASH practice and stunting among preschool children

Table 2 shows the WASH status of the households in this study. The majority (299, or 72%) of the 415 households used an unimproved toilet (a pit latrine without a slab). One out of five households had no toilet facilities. Moreover, 159 (38% of households) owned nonfunctional toilet facilities. Almost four out of every five households had no hand washing facilities near the toilets during the survey. From the study respondents, two hundred forty-six (59.3%) did not dispose of children’s feces in latrines. On the other hand, from a total of 415 households interviewed about drinking water, only 146 (35.2%) used unimproved water, did not get improved water below 30 minutes for a round trip, 287 (69.2%) got less than 20 l per day per person, and 239 (57.6%) of them were found above 1 km from water sources. Mothers and caregivers washed their hands at critical times: after defecation, after cleaning babies’ anuses, before eating or feeding children. Based on this, 301 (72.5%), 174 (41.9%), and 248 (40.2%) did not wash their hands after defecation or after cleaning their babies’ anus before eating or feeding, respectively (See Table 2).

**Table 2:** Environmental condition and hand washing practices of residents of Kebeles in Lemo district (*n* = 415)

Variables	Frequency	percent
**Does household have toilet**		
Yes	324	78.1
No	91	21.9
**Toilet with hand washing**		
Yes	74	17.8
No	341	82.2
**Safe water management**		
Yes	415	100
No		
**Toilet Condition**		
Functional	256	61.7
Not functional	159	38.3
**Hand washing after defecation**		
With water and soap	114	27.5
With water only or does not wash	301	72.5
**Hand washing after cleaning child’s anus**		
With water and soap	241	58.1
With water only	166	40
Does not wash totally	8	1.9
**Hand washing before eating/ feeding childes**		
With water and soap	248	59.8
With water only	137	33
Does not wash totally	40	9.6

### Factors associated with stunting among children

Based on the report in Table 3, seven variables were found to be associated with stunting in the study. However, only six (6) variables showed a statistically significant association with stunting. Except for the variable of household members who get less than 20 l of water, the other variables were statistically significant on the multivariate logistic regression analysis model. These include the following variables: Children whose mothers threw their children’s feces in the open field; children living in households with unimproved latrines; children living in families with unimproved water sources; children of women who did not wash their hands with water and soap; and children of uneducated women Children aged 36–47 months were 2.8 times more likely to be stunted compared to children aged 24-36 months (AOR = 2.8, 95% CI, 1.1, 7.1). Stunting was 2.8 times more likely in children aged 48-59 months than in children aged 24-36 months (AOR = 2.8, 95% CI: 2.1, 12.1). Stunting occurs 1.8 times more frequently in children of uneducated mothers than in their counterparts (AOR = 1.8, 95% CI: 1.14-2.76; Children who lived in households with unimproved toilets were 3.6 times more likely to be stunted than their counterparts (AOR = 3.6, 95% CI, 1.43, 9.03). The risk of stunting was 2.8 times higher in children of mothers who had not properly disposed of their child’s feces (AOR = 2.8, 95% CI, 1.6, 5.3). The odds of stunting among children whose mothers did not wash their hands with water and soap before eating or feeding their children were 1.7 times higher than their counterparts (AOR = 1.7, 95% CI, 1.1, 2.6). Children whose mothers did not wash their hands before feeding their children had a higher risk of stunting in the study area (AOR = 6.2, 95% CI: 2.1, 19.1) (See Table 3).

**Table 3:** Multivariable logistic regression analysis of factors associated with stunting among under five children in Lemo district South Ethiopia, 2019 (415)

Variables	Stunted	Normal	Crude (95% CI)	Adjusted (95% CI)
Childs age(months)				
24-35	69	38	1	1
36-47	97	52	1.98 (1.2,3.3)*	1.6 (1.1, 7.1)**
48-59	113	46	3.9 (2.3,6.7)*	2.8 (2.1,12.1)**
Mother education				
Educated	19	225	1	1
Uneducated	26	145	2.1 (1.13, 3.97)*	1.8(1.5, 4.2)**
Types of toilets				
Improved	149	87	1.5(1.02, 2.24)*	1.7(1.2,2.6)**
Unimproved	95	84	1	1
Child feces disposal				
Safely	134	34	1	1
Unsafely	142	105	2.9(1.9,4.6)**	2.7(1.6,5.3)**
Hand washing with soap before feeding child				
Yes	236	157	2.6(1.07, 6.41)*	6.2 (2.0, 19.1)**
No	8	14	1	1
Hand washing with soap after defecation				
Yes	23	215	1	1
No	40	137	2.7(1.6,4.7)*	3.1(1.3,8.3)**
Water per day/person/litters				
> 20 Litters	44	200	1	1
< 20 Litters	19	152	0.46 (0.25, 0.84) *	1.13 (0.53, 2.40)

*Significant with *P*-value< 0.05, **Significant with *P*-value < 0.001

## Discussion

This study revealed that the prevalence of stunting or chronic malnutrition among children of age 24 to 59 months was found to be 33.5% (95%CI = 31.2, 35.8%) in current study area. This finding is lower than the national prevalence of stunting in similar study population group in Ethiopia which account as 38% [22]. The possible difference might be associated with the fact that the national finding might be more reliable due to large sample size.

This finding is lower than findings of studies done in different local areas in Ethiopia: Gurage zone, South Ethiopia (52.5%), Albuko, Northeast Ethiopia (39.3%), Gojam, and Northwest Ethiopia (37.5%). Besides, this finding is also lower than finding of local area in another country: Bangladesh (41% )[23–26]. This might be due to difference in study setting, level of awareness of the care giver and accessibility of health service. This finding is more than findings of other studies done in Sodo zuria district, South Ethiopia (24.9%), South East Kenya (23.3%), West Guji, South Ethiopia (31.8%) [27–29]. This might be due to difference in socio-demographic and some contextual factors of the study setting. Moreover, the current study area is one of the food insecure areas in Ethiopia.

The results of this study show a strong correlation between stunting and child age. Stunting is more prevalent as people get older. This result is in line with research from Gojam [29] and Rwanda [30]. Inappropriate food supplementation during the weaning stage, when infants should move from exclusively breastfeeding to include supplementary meals in their diet, may be the cause of the steady increase in stunting among children aged 24 to 59 months [22, 31, 32]. Children whose mothers or other primary caregivers have received breastfeeding and complementary feeding education through the Village Community Based Nutrition Program [30]. However, because this is a self-selecting group that chooses to attend these educational sessions, mothers of malnourished or at-risk children may not be included. Mothers may also lengthen the duration.

In contrast, the current finding conflicts with findings from Indonesia [29] and national EDHS studies [21]. In utero stunting is a cumulative process that can last up to three years after delivery. The results of this investigation revealed that the risk of stunting increases with age, which is not surprising. The study’s findings indicate that the first two years of life are the most crucial time for intervention, and it is urgently necessary to start initiatives that will help the most vulnerable children in the study region in terms of their nutritional health. When implemented among children in the first three years of life, such programs are likely to be the most beneficial. Youngsters under 24 months old reacted to the change considerably more quickly than children who were older.

In comparison to their peers, children of uneducated women were more likely to be stunted [29]. This may be connected to the fact that children’s health and nutritional status can be impacted by their schooling. Because it gives women access to modern healthcare and the knowledge and skills they need to take care of children [30]. Children who lived in homes with older toilets had a higher likelihood of being stunted than their counterparts [31, 32]. Since poor homes are less likely to have sanitary toilet facilities, the type of a household’s sanitary facilities is a good indicator of the household’s wealth. The findings of Dearden, K. A., et al. (2017) and Khatab, K. (2010) [32, 33] are consistent with this finding. Children in household which had toilet without hand washing service had more chance to be stunted than their counterparts [30, 31]. This might be explained with the fact that open defecation free status had lower prevalence of diarrhoea cases compared open defecation. This suggests diarrhoea is associated with stunting [32]. Children whose faeces were disposed in open field defecation were more likely to be stunted than their counterparts [33]. This might be associated with lack of access to toilets and other sanitation-related facilities; the practice of open defecation poses severe threats to the overall health of populations, particularly children of the developing countries, through various linkages.

Children whose mother does not wash hand after defecation were more likely to be stunted than their counterparts. This finding is consistent with the previous study conducted in Indonesi a[34] and rural Indi a[34]. This might be explained due to the fact that he evidence reviewed suggests that poor WASH conditions have a significant detrimental effect on child growth and development resulting from sustained exposure to enteric pathogens but also due to wider social and economic mechanisms [35].

### Limitation

This study had a number of strengths. It provides a more comprehensive overview of the prevalence and factors associated with stunting (primarily WASH factors) in this food-insecure area, with different feeding styles, WASH practices, and coping mechanisms for food insecurity. The random selection, minimum non-response rate, and adequate sample size of the study subjects make the findings of this study more generalizable. However, this study cannot address some factors associated with food availability and accessibility that affect stunting.

### Public health implication

The findings of this study indicated that food security programs should extensively incorporate water, personal hygiene, and environmental sanitation conditions into an effective strategy for food security programs.

## Conclusion

In conclusion, the prevalence of stunting among children aged 24 to 59 months is high in this study area. Increased child age, more maternal education, higher household income, unimproved toilets, and a lack of hand washing among mothers of children before feeding a child were predictors that increased stunting among children aged 6 months to 59 months. Environmental sanitation and personal hygiene programs should be strengthened. Furthermore, the socio-economic status of women should be improved.

## Data Availability

The datasets used and/or analyzed during the current study available from the corresponding author on reasonable request.

## Abbreviations

AOR: Adjusted Odds ratio
COR: Crude Odds Ratio
ODF: Open Defecation Field
CI: Confidence Interval
WHO: World Health Organization
